# The Trickle-Down Effect of Leaders’ VWGB on Employees’ Pro-Environmental Behaviors: A Moderated Mediation Model

**DOI:** 10.3389/fpsyg.2021.623687

**Published:** 2021-04-21

**Authors:** Jianfei Wu, Weinan Zhang, Chuanhu Peng, Juan Li, Saiyu Zhang, Wenjing Cai, Dan Chen

**Affiliations:** ^1^Ance Think Tank, Hefei, China; ^2^School of Public Affairs, University of Science and Technology of China, Hefei, China; ^3^Department of Management and Organization, Vrije Universiteit Amsterdam, Amsterdam, Netherlands; ^4^International Institute of Finance, School of Management, University of Science and Technology of China, Hefei, China

**Keywords:** green self-identity, leaders’ voluntary workplace green behavior, proactive pro-environmental behaviors, task-related pro-environmental behaviors, trickle-down effect

## Abstract

Although previous research has highlighted the positive effect of leaders’ voluntary workplace green behavior (VWGB), limited research attention has been given to empirically testing how and when such behavior produces trickle-down effects. Taking a role model perspective and drawing on social identity theory, this research aims to fill this gap by proposing and testing the mechanism and boundary conditions of the influencing processes whereby leaders’ VWGB can trickle down to employees’ pro-environmental behaviors. By theorizing a moderated mediation model, the current research empirically examines the hypotheses by conducting a hierarchical regression analysis. We employed a survey questionnaire research design to collect two waves of multisource data. The data used in the analysis are from survey responses of 313 subordinate-supervisor dyads at two different time points. The results show that leaders’ VWGB can have a trickle-down influence on employees’ task-related pro-environmental behavior and proactive pro-environmental behaviors through their green self-identity and that this trickle-down effect is greater among employees with higher green climate perceptions. Our results reveal the intervening mechanism and boundary condition of leaders’ VWGB by conducting a systematic examination of how this effect trickles down.

## Introduction

The ongoing struggle of all organizations in the modern business world to achieve long-term success and thrive has highlighted the implications of green management throughout organizations (Piening and Salge, 2015; Albort-Morant et al., 2018). This trend, accordingly, has gradually attracted both researchers and practitioners, who highlight the critical endeavors aiming to improving pro-environmental behaviors among staff (Robertson and Barling, 2013; Afsar et al., 2016; Peng et al., 2020) because they are responsible for green tasks and duties in the workplace. This research direction indicates that green management is becoming increasingly important for the long-term development of the service industry, especially in the tourism sector, since a win-win ‘green and competitive’ strategy can help tourism organizations (e.g., hotels) to achieve competitive advantages, innovation, and customer loyalty and trust (Chou, 2014; Zhang and Huang, 2019). Given this significance, the existing literature has identified a wide array of predictors of green outcomes among employees (Kollmuss and Agyeman, 2002; Han et al., 2009; Norton et al., 2015). Among them, scholars have provided accumulated evidence to show that leadership plays a key role in sustainable processes in the workplace because the direct supervisor represents a critical component of the social structure in organizations and acts as a vital agent of the firm, based upon whom followers develop their judgments of their working companies (Volmer et al., 2012).

Recently, scholars have pointed out that *leaders’ voluntary workplace green behavior (VWGB)*—referring to leaders’ discretionary activities and behaviors that aim to enhance organizational sustainability in a green and environmental way but that are not regulated or controlled by formal institutions relating to green managerial philosophy (Boiral, 2009)—can influence followers’ desirable outputs (Kim et al., 2017; Cai et al., 2020). However, the empirical attention given to the trickle-down effect of leaders’ VWGB is still very limited. In the current study, we attempt to fill this research gap by investigating *why, how, and when leaders’ VWGB trickles down to influence employees’ relevant work behaviors*.

Scholars in the pro-environmental literature have contended that leaders’ green behaviors or environmental leadership styles may trigger desirable outcomes among followers through identity mechanisms (Tian and Robertson, 2019; Peng et al., 2020). Based on the theoretical framework of social identity theory, situations can influence an individual to identify with their group (e.g., organization) through social classification (Turner and Oakes, 1986), which in turn drives their attitudes and behaviors (Meal and Ashforth, 1989); that is, when the leader can first adjust the relevant part of the employee’s self-identity or self-concept, which originates from his/her (i.e., employees’) cognition and emotional attachment to team membership, would these employees’ behaviors be adjusted correspondingly (Tajfel, 1974). In relation to the green literature, this theory implies that when employees perceive their leaders engaging in such behaviors in a pro-environmental manner, their self-identity regarding green behaviors is likely to be more strongly shaped because followers who see themselves as a part of a particular unit tend to make their green self-identity consistent with their leader’s equivalent behaviors. Moreover, since employees’ work behaviors are typically developed in the workplace, their psychological relationship with the group’s green values, as formed by their leaders, may affect their motivation to exert environmental effort. Specifically, leaders who are normally treated as representatives of the organization play a central role in the evolution and cultivation of the organization in terms of its internal working environment; that is, because the status of the leader is higher than that of an employee’s coworkers, we suggest that the green-oriented behavior of the leader can significantly directly influence that employee’s green-related self-identity. Therefore, we propose that an employee’s green self-identity is a mediator of the trickle-down effect of leaders’ VWGB on employees’ pro-environmental behaviors.

**Figure 1:**
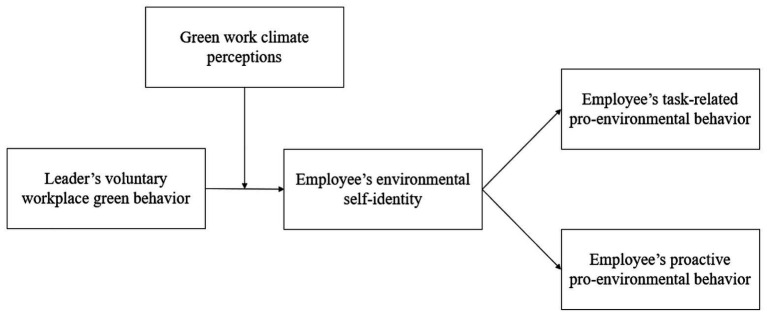
The hypothesized model.

Prior literature has specified that employees’ perceptions of their work climate is linked to the extent to which they can access practical support for their attempts to behave in an environmental manner (Smith-Crowe et al., 2003). In this vein, we further propose that employees’ perceptions of the green climate — defined as “employees’ perceptions and interpretations of their organization’s policies, procedures, and practices regarding environmental sustainability” (Norton et al., 2015, p. 212) — in their organization can act as a boundary condition that specifically strengthens the trickle-down impact of leaders’ VWGB on followers’ green endeavors (e.g., pro-environmental behaviors) *via* their green self-identity. Specifically, when employees work in a green-oriented organization, they are more likely to persistently enact pro-environmental behaviors that are highly aligned with the organization’s environmental performance goals (Chou, 2014) because they sense that their pro-environmental behaviors are highly valued and encouraged by the organizations (Dumont et al., 2017). Thus, employees are more likely to follow their leaders’ VWGB to develop their green self-identity and, subsequently, pro-environmental behaviors. Figure 1 shows the hypothesized model.

Our current study attempts to contribute to the existing green literature in the following ways. First, the study theoretically proposes and empirically tests the trickle-down influence of leaders’ VWGB in organizations. In doing so, we contribute to the existing literature by conceptualizing environmental behaviors at the leader level as a way of modeling a specific work behavior (i.e., VWGB) for followers to effectively boost pro-environmental behavior at the employee level. Second, by exploring the mechanism behind this influence, this study reveals that leaders’ VWGB trickles down to influence green-oriented outcomes such as pro-environmental behaviors among followers by boosting employees’ environmental self-identity. Although supervisors are essential for conveying green awareness to other staff in the workplace, there are limited research efforts exploring the underlying psychological processes that may help supervisors to endorse inclusion efforts throughout the company. Finally, by identifying employees’ green climate perceptions, we make a unique contribution by investigating situational factors that are part of the process through which leaders’ VWGB influences employees’ green self-identity and pro-environmental behaviors.

## Theoretical Background and Hypotheses Development

### Leaders’ VWGB and Employees’ Green Self-Identity

The construct of VWGB has its origins in the green and environmental literature and corresponds to researchers’ recent call for exploring appropriate leader behaviors in the workplace that can contribute to the growth of desirable outputs from followers such as green innovation (Fernández et al., 2006; Goldstein et al., 2008). Specifically, *leaders’ VWGB* has been widely acknowledged to be a beneficial predictor. When a leader voluntarily displays green-oriented behaviors in the workplace, his/her followers receive information that environment-related endeavors are considered to be valuable and encouraged; thus, followers are more likely to engage in green-oriented behaviors during their work (e.g., green innovation, sustainable activities; Kim et al., 2017, 2019; Cai et al., 2020). The theoretical basis for the benefits of leaders’ VWGB in facilitating desirable outputs among followers lies in the tendency of subordinates to form and develop their own behavioral styles by observing, identifying, and imitating the behaviors of leaders (Bandura and Walters, 1977; Babutsidze and Chai, 2018). For example, Chan (2011) found that when working under a green-oriented leader, employees may generate an interpretive cognition of the leader’s environmental management and protection and thereby attribute meaning to individual environmental activities during working hours (Chan, 2011). Following this line of argument, we draw on social identity theory to propose that leaders’ VWGB can exert a positive influence on followers’ green self-identity.

Defined as how individuals describe themselves in terms of their social interactions and expectations about related others (Ellemers et al., 2002), green self-identity helps individuals advocate the morals and actions of a specific group of individuals to which they would like to belong or feel like they already belong (van Gils and Horton, 2019). In the current study, we expect leaders’ VWGB to influence employees’ green self-identity because personal identity originates in the contextual expectations of others and the multiple roles that people hold (Tajfel, 1974; Stryker and Burke, 2000; Ellemers et al., 2002). When leaders display voluntary green behaviors, employees may receive from those leaders the information that eco-friendly behaviors should be reinforced because the significance of green endeavors in the workplace is highlighted and valued. In this situation, employees tend to describe their green orientation in terms modeled by their leaders (Afsar et al., 2016; Mittal and Dhar, 2016). This categorization of a green orientation can be seen as a typical attribute of the work group (Lord and Brown, 2003). Consequently, employees’ self-identification with engagement in green endeavors would be enhanced. Thus, we hypothesize as follows:

*Hypothesis 1: Leaders’ VWGB is positively related to employees’ green self-identity.*

### Employees’ Green Self-Identity and Pro-Environmental Behaviors

Social identity theory suggests that instead of being a passive recipient, an individual can be an active agent in the creation of his/her sense of self-identity (Meal and Ashforth, 1989; Ellemers et al., 2002). Accordingly, we propose a positive influence of employees’ self-identity on their pro-environmental behaviors. Conceptually, green self-identity refers to a specific label used to describe oneself (Cook et al., 2002); when an individual identifies himself/herself as a part of a specific group, he/she tends to enact certain behaviors that are in line with the behaviors within that group (Terry and Hogg, 1996; Ellemers et al., 2002). Related research with similar results can be found within the green literature (Whitmarsh and O’Neill, 2010; Chang and Chen (2013); Peng et al., 2020). For example, Confente et al., 2020 found that customers with a green self-identity place a high perceived value on green products, which leads to stronger behavioral intentions.

Employees’ pro-environmental behaviors can be divided into *task-related pro-environmental behavior* and *proactive pro-environmental behavior* (Utterback and Abernathy, 1975; Albort-Morant et al., 2018). Specifically, task-related pro-environmental behavior is defined as personal specific behaviors of completing required responsibilities and jobs by employing environmental, green, and friendly approaches, while proactive pro-environmental behavior is defined as individuals’ proactive initiatives enacting environmental, green, and friendly behaviors beyond the realm of their required responsibilities and job tasks. Theoretically, task-related pro-environmental behavior highlights the completion of expected core work tasks (or in-role behaviors; Williams and Anderson, 1991) involving the preservation of natural resources and protection of the environment. Proactive pro-environmental behavior focuses on personal initiative and an active, self-initiating work approach that is not formally required for the job. These two types of pro-environmental behaviors are distinct but related forms of workplace behavior. Both concepts refer to employees’ discretionary environmentally friendly actions in the workplace.

We propose that employees’ green self-identity is positively related to their task-related and proactive pro-environmental behaviors. Specifically, employees with a green self-identity may conform to the green values, beliefs, and behaviors of work teams supervised by green leaders (Christensen et al., 2004). In this situation, employees may attempt to establish consistent green attitudes and actions with continuity across experiences; thus, green self-identity appears to be highly relevant in exploring consistency (and, ultimately, spill-over effects) across pro-environmental behaviors. Moreover, a green self-identity gives a strong signal that engaging in green behaviors is consistent with employees’ group identification (Whitmarsh and O’Neill, 2010; Chang and Chen, 2013), acting as a significant determinant of green behaviors because employees highly identify with engaging in pro-environmental activities.

Combining the hypotheses and theoretical arguments above, we propose a mediating effect of employees’ green self-identity: specific leader behaviors that exert more positive effects on employees’ relevant self-identity will have a stronger influence on followers’ specific behaviors. Specifically, when employees see their leaders voluntarily engage in green behaviors, they are motivated to identify as green in the workplace because they already identify themselves as green agents. As a result, they behave in a more environmentally friendly way. Accordingly, we propose two hypotheses as follows:

*Hypothesis 2a: Employees’ green self-identity mediates the relationship between leaders’ VWGB and employees’ task-related pro-environmental behaviors.*
*Hypothesis 2b: Employees’ green self-identity mediates the relationship between leaders’ VWGB and employees’ proactive pro-environmental behaviors.*

### Employees’ Green Climate Perception as a Boundary Condition

Past research findings have argued that the impacts of leaders’ supervisory behaviors are differentiated by contextual factors (Kerr and Jermier, 1978; Podsakoff et al., 1993), such as the organizational environment (Ling et al., 2016). Thus, in the current research, we propose that employees’ green climate perception serves as a boundary condition in the process by which leaders’ VWGB influences followers’ environmental self-identity. Theoretically, employees’ perception of their organizational work environment reflects personal value-based schemas that aim to explain various information in the workplace (James et al., 2008), espoused values, and behavioral norms (Clegg and Spencer, 2007), and these can further influence employees’ corresponding behaviors (Feng and Chen, 2018). Accordingly, employees’ green climate perception reflects their recognition and interpretation of organizational strategies and practices in terms of the environmental and green philosophy (Norton et al., 2015). As a collective phenomenon, the green climate in organizations can regulate employees’ environmentally oriented attitudes and behaviors by giving them guidelines and goals directed towards desired outcomes (Schneider and Reichers, 1983).

We expect that the perception of the green work climate among employees can moderate the association between leaders’ VWGB and employees’ environmental self-identity. Specifically, the effectiveness of leaders’ VWGB is expected to be more strongly correlated with employees’ environmental self-identity in situations where the employees perceive the organization’s work environment as green and environmental. Since the theoretical arguments suggest that the green climate represents the collective norms and values of green-relevant endeavors, employees working in a high-level green climate have a greater potential of being more green oriented (Dumont et al., 2017). That is, when they perceive their work climate as green, employees abide by the organization’s regulations and participate in developing their green-relevant attitudes and behaviors (Norton et al., 2017; Zientara and Zamojska, 2018). Furthermore, a green and environmental perception of the work climate signals that a green orientation (e.g., an environmental mindset and behaviors) is valued, encouraged, and even rewarded by the organization (Chou, 2014). In this situation, employees’ attention is directed towards their leaders’ behaviors, such as VWGB, as they strive to self-identify with environmental activities.

In contrast, when employees are working in an organization that has less concern about creating a green work climate, they naturally express less desire to work in an environmental way because they sense that their efforts should be directed towards other areas rather than towards green-relevant issues. They thus ignore the VWGB of their leader, as his/her green activities are not in step with organizational values. In such a context, employees’ attention fails to be directed towards learning and following their leaders’ VWGB, as they feel that an environmental orientation is not highly encouraged by their organizations (Chou, 2014). Moreover, a weak green work climate may cause employees to be insensitive to green-relevant issues because such issues are not targeted by the organization or their coworkers (Dumont et al., 2017). That is, it is difficult for these employees to observe their leaders’ VWGB. Consequently, employees are less likely to develop an environmental self-identity. Taking the aforementioned theoretical arguments and reasoning, we propose the third hypothesis in the current study:

*Hypothesis 3: Employees’ green climate perception positively moderates the relationship between leaders’ VWGB and employees’ green self-identity such that the positive relationship between leaders’ VWGB and employees’ green self-identity will be stronger when employees’ green climate perception is high.*

Overall, we expect a moderated mediation model: the conditional indirect impact of leaders’ VWGB on employees’ multiple pro-environmental behaviors in the workplace (i.e., task-related and proactive pro-environmental behaviors) through employees’ green self-identity will be stronger when employees perceive the organization as having a strong green climate. That is, when employees perceive that their organizational climate supports green issues and affairs, it is more likely that they will recognize green-relevant activities in their daily work. In this situation, they tend to emulate their leaders’ display of VWGB in developing their self-identity around environmental concerns; therefore, they significantly increase both task-related and proactive pro-environmental behaviors. In contrast, in conditions where the work environment does not emphasize green issues, employees may perceive that engaging in pro-environmental activities is not highly valued. As a result, a leader’s display of VWGB will not necessarily encourage followers to be environmental in the workplace, as followers will potentially prioritize non-green activities over developing their environmental self-identity and pro-environmental behaviors. On this basis, the last two hypotheses are proposed as follows:

*Hypothesis 4a: Employees’ green climate perception moderates the indirect effect of leaders’ VWGB on employees’ task-related pro-environmental behaviors through employees’ green self-identity, such that the indirect effect becomes stronger when employees’ green climate perception is high.*
*Hypothesis 4b: Employees’ green climate perception moderates the indirect effect of leaders’ VWGB on employees’ proactive pro-environmental behaviors through employees’ green self-identity, such that the indirect effect becomes stronger when employees’ green climate perception is high.*

## Materials and Methods

### Sampling and Procedure

We conducted a survey design to collect two-source data from 10 hotels in central provinces in mainland China. These 10 hotels were all high star-level hotels that were invited to participate in a project on improving the service of hotels in Anhui Province, P.R. China, and this invitation included various departments (e.g., front office, housekeeping, food and beverage, recreation, and security). These hotels were located in urban areas with special naturalistic-environmental interest. Before collecting data, one of the authors contacted the hotel managers and informed them of the research topic. After receiving their confirmation that the hotels were currently in favor of environmentally friendly activities and that they were willing to participate in our research, we asked the human resource department in each hotel to invite interested employees to fill in the questionnaires voluntarily. Specifically, the hotels that participated in our current study all implemented environmental quality policies to improve the quality of their environmental services. For example, saving energy (e.g., saving water and recycling papers) was part of employees’ job descriptions. A total of 515 employees indicated their willingness to join in our research, and they agreed to complete the questionnaires during their working hours.

A time-lagged research design was used; that is, we collected the survey data in two rounds, separated by a one-month time interval to avoid the possibility of common method bias (N. Podsakoff, 2003). At Time 1, we submitted the first questionnaires to 515 employees and asked them to rate their perception on their direct leaders’ VWGB and provide their perception of the green climate in their organization, their green self-identity and some demographic information. After removing incomplete questionnaires, 432 valid questionnaires remained. We asked the HR department for assistance in matching pairs of 432 employees and their direct supervisors. One month later, at Time 2, we submitted the questionnaires to these employees’ direct supervisors (i.e., *N* = 68) to rate these employees’ pro-environmental behaviors as perceived by their leader. We received 60 supervisor responses; that is, the final matched sample consisted of 313 employees and 60 supervisors, with 5.2 employees per supervisor on average. Among these employees, the average age was 28.88 years old, 66.1% of the participants were female, most of them had an associate degree (55.0%), and their average tenure in the current organizations was 3.78 years.

### Measures

All survey items were from previous research and have been widely utilized in the pro-environmental literature. We followed the back-translated procedure from Brislin (1980) to create the measures in the Chinese version. Unless otherwise noted, a response format of 1 = “strongly disagree” to 5 = “strongly agree” was employed to rate the measures.

#### Leaders’ VWGB as Perceived by Employees

We used a scale with six items from Kim Kim et al. (2017) to rate employees’ perception of their leaders’ VWGB. The items are “My supervisor recycles reusable things in the workplace,” “My supervisor avoids unnecessary printing to save paper,” “My supervisor uses personal cups instead of disposable cups,” “My supervisor uses stairs instead of elevators when going from floor to floor in the building,” “My supervisor reuses paper to take notes in the office,” and “My supervisor sorts recyclable materials into their appropriate bins when other group members do not recycle them” (Cronbach’s *α* = 0.93).

#### Employees’ Green Self-Identity

A scale containing three items developed from Van der Werff et al. (2014) was used to rate employees’ green self-identity. The items are “I am the type of person who acts pro-environmentally,” “Acting pro-environmentally in an important part of who I am,” and “I see myself as a pro-environmental person” (Cronbach’s *α* = 0.91).

#### Employees’ Green Climate Perception

We used the 4-item scale from Norton et al. (2012) to assess employees’ green climate perception. The items are “Our hotel is worried about its environmental impact,” “Our hotel is interested in supporting environmental causes,” “Our hotel believes it is important to protect the environment,” and “Our hotel is concerned with becoming more environmentally friendly” (Cronbach’s *α* = 0.86).

#### Task-Related Pro-Environmental Behavior as Perceived by Leaders

We used Bissing-Olson et al. (2013) scale with three items to assess employees’ task-related pro-environmental behavior as perceived by their leaders. The items are “He/she fulfilled responsibilities specified in his/her job description in environmentally friendly ways,” “He/she adequately completed assigned duties in environmentally friendly ways,” and “He/she performed tasks that are expected of me in environmentally friendly ways” (Cronbach’s *α* = 0.85).

#### Proactive Pro-Environmental Behavior as Perceived by Leaders

We used Bissing-Olson et al. (2013) scale with three items to assess employees’ proactive pro-environmental behavior as perceived by their leaders. The items are “He/she took a chance to get actively involved in environmental protection at work,” “He/she took initiative to act in environmentally friendly ways at work,” and “He/she did more for the environment at work than he/she was expected to” (Cronbach’s *α* = 0.89).

#### Control Variables

Some variables were controlled in the study: age (in years), gender (1 = male; 2 = female), education level (1 = junior high school diploma and below, 2 = high school/technical school diploma, 3 = Associate degree, 4 = Bachelor’s degree, 5 = Master’s degree and above), and working years in the current hotel (in years).

## Results

### Validity Test

Before testing the hypotheses, we used AMOS to conduct confirmatory factor analyses (CFA) to validate the fit of our hypothesized model. Table 1 shows the results. Specifically, our hypothesized five-factor model revealed a better fit to the data (*χ*^2^ = 531.44; *df* = 291; TLI = 0.97; CFI = 0.97; RMSEA = 0.05) than all the alternative models (Hu and Bentler, 1999).

**Table 1:** Results of CFA.

Models	*χ*^2^	*df*	TLI	CFI	RMSEA
Hypothesized four-factor model	531.44	291	0.97	0.97	0.05
Three-factor model (green work climate perception and employees’ environmental self-identity combined)	766.28	299	0.80	0.80	0.11
Two-factor model (leaders’ VWGB, green work climate perception and employees’ environmental self-identity combined)	1138.34	301	0.76	0.77	0.14
One-factor model (all combined)	1390.21	303	0.62	0.62	0.18

Table 2 shows the means, standard deviations, and correlations of the variables in the study. Consistent with our expectation, leaders’ VWGB as perceived by employees is positively correlated with both employees’ green self-identity (*r* = 22, *p* < 0.01) and employees’ pro-environmental behaviors as perceived by their leader (task-related pro-environmental behavior *r* = 0.21, *p* < 0.01; task behavior *r* = 0.29, *p* < 0.01).

**Table 2:** Means, standard deviations, and correlations.

Variables	Mean	SD	1	2	3	4	5	6	7	8
1. Gender	1.67	0.48								
2. Age	28.88	5.83	0.06							
3. Education	3.05	0.83	0.01	−0.28**						
4. Tenure	3.78	3.65	0.06	0.50**	−0.20**					
5. Leaders’ VWGB	3.36	1.06	−0.01	−0.02	0.06	−0.01				
6. Employees’ green self-identity	2.06	1.01	−0.06	−0.04	0.05	−0.04	0.22**			
7. Green climate perception	4.24	0.98	0.02	0.04	0.04	0.04	0.26**	0.36**		
8. Task-related pro-environmental behavior	3.92	1.02	0.01	0.01	0.00	0.04	0.21**	0.25**	0.63**	
9. Proactive pro-environmental behavior	4.29	1.19	0.04	−0.02	0.06	0.00	0.29**	0.27**	0.56**	0.76**

*N* = 313. ^*^p < 0.05; ^**^p < 0.01; ^***^p < 0.001

### Hypotheses Testing

We used a simple regression to test Hypothesis 1, which proposes the positive relationship between leaders’ VWGB as perceived by employees and employees’ green self-identity. In Table 3, the results in Model 1 indicate that leaders’ VWGB as perceived by employees is positively related to employees’ green self-identity (*β* = 0.24, *SE* = 0.03, *p* < 0.001), supporting Hypothesis 1. In Model 2, employees’ green self-identity is positively related to their task-related pro-environmental behavior (*β* = 0.69, *SE* = 0.05, *p* < 0.001), but leaders’ VWGB as perceived by employees is not significantly related to employees’ task-related pro-environmental behavior (*β* = 0.03, *SE* = 0.04, *p* > 0.05); that is, employees’ green self-identity fully mediates the relationship between leaders’ VWGB as perceived by employees and employees’ task-related pro-environmental behavior as perceived by their leader. Thus, Hypothesis 2a is supported. In Model 3, both leaders’ VWGB (*β* = 0.11, *SE* = 0.04, *p* < 0.05) and employees’ green self-identity (*β* = 0.88, *SE* = 0.05, *p* < 0.001) are positively related to employees’ proactive pro-environmental behavior as perceived by their leader; that is, employees’ green self-identity partially mediates the association between leaders’ VWGB as perceived by employees and followers’ proactive pro-environmental behaviors as perceived by their leader. Thus, Hypothesis 2b is supported.

**Table 3:** Results of simple regression.

Variables	Model 1 Employees’ green self-identity		Model 2 Task-related pro-environmental behavior		Model 3 Proactive pro-environmental behavior		Model 4 Employees’ green self-identity	
	*β*	*SE*	*β*	*SE*	*β*	*SE*	*β*	*SE*
Gender	0.05	0.11	−0.01	0.09	0.06	0.09	−0.02	0.08
Age	0.01	0.01	−0.01	0.01	−0.01	0.01	0.01	0.01
Education	0.05	0.07	−0.05	0.05	0.02	0.06	0.09	0.05
Tenure	0.01	0.02	0.01	0.01	−0.00	0.01	0.00	0.01
Leaders’ VWGB	0.24^***^	0.05	0.03	0.04	0.11^*^	0.04	−0.21^**^	0.07
Employees’ green self-identity			0.69^***^	0.05	0.88^***^	0.05	−1.12^***^	0.10
Leaders’ VWGB × Employees’ green climate perception							0.15^***^	0.03
Δ R^2^	0.06		0.45		0.58		0.04	
Δ F	21.51		127.84		211.80		23.67	

*N* = 313.

* *p* < 0.05;

** *p* < 0.01;

*** *p* < 0.001.

**Figure 2:**
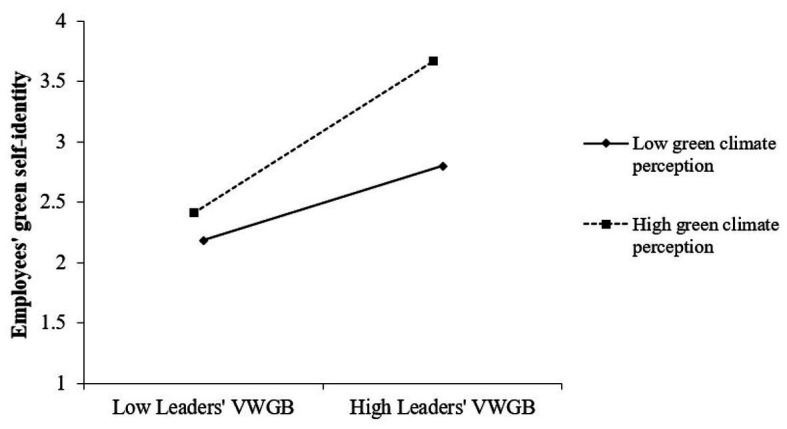
The moderating effect of green climate perception on the relationship between leaders’ VWGB and employees’ green self-identity.

Regarding testing Hypothesis 3, we computed an interactive term (i.e., the variable of Leaders’ VWGB × Employees’ green climate perception) in a two-way interaction and entered it into the regression. As the results show in Model 4, the interaction term is positively related to employees’ green self-identity (*β* = 0.15, *SE* = 0.03, *p* < 0.001). To further test Hypothesis 3, we then followed Aiken et al. (1991) to depict the pattern of the interaction effect. The simple slope test is presented in Figure 2. The figure indicates that employees’ green climate perception significantly strengthened the positive influence of leaders’ VWGB on employees’ green self-identity when the latter was high rather than low. Taking these findings together, Hypothesis 3 is fully supported.

To test the moderated mediation hypotheses (i.e., Hypotheses 4a and 4b), we used the SPSS PROCESS macro (Hayes, 2013). The results in Table 4 indicate that the impact of leaders’ VWGB as perceived by employees on employees’ task-related pro-environmental behavior *via* employees’ green self-identity was strengthened when green climate perception was added as a moderator (*β* = 0.11, *SE* = 0.02; LCI = 0.06, UCI = 0.16). Similarly, the trickle-down influence of leaders’ VWGB on employees’ proactive pro-environmental behavior *via* employees’ green self-identity was strengthened when green climate perception was added as a moderator (*β* = 0.14, *SE* = 0.03; LCI = 0.08, UCI = 0.20). Probing the conditional indirect effects at 1 *SD* above and below the mean supports these propositions and further establishes that the effects are significant only when green climate perception is high. Thus, as expected, higher levels of green climate perception positively moderate the mediation relationship between leaders’ VWGB, employees’ green self-identity, and both task-related and proactive pro-environmental behaviors as perceived by their leader. Both Hypothesis 4a and 4b are supported.

**Table 4:** Results of conditional indirect effects.

Relationship between leaders’ VWGB and task-related pro-environmental behavior *via* employees’ green self-identity			
Index	*SE*	LCI	UCI
0.11	0.02	0.06	0.16
Moderator (green climate perception)	Indirect effect	SE	95% CI
Low levels of moderator (−1 SD)	−0.04	−0.08	0.00
High levels of moderator (+1 SD)	0.20	0.11	0.30
**Relationship between leaders’ VWGB and proactive pro-environmental behavior *via* employees’ green self-identity**			
Index	*SE*	LCI	UCI
0.14	0.03	0.08	0.20
Moderator (green climate perception)	Indirect effect	SE	95% CI
Low levels of moderator (−1 SD)	−0.05	−0.11	0.00
High levels of moderator (+1 SD)	0.25	0.14	0.38

*N* = 313. 5,000 bootstraps (95% confidence intervals). *SE*, standard error; LCI, lower confidence interval; UCI, upper confidence interval.

## Discussion

### Theoretical Implications

By theorizing the trickle-down influence of leaders’ VWGB as perceived by employees on the two types of green-oriented work behaviors among employees (i.e., task-related and proactive pro-environmental behaviors) through the mechanism of employees’ green self-identity and conditioned on green climate perceptions, we aim to provide several theoretical contributions to the current green research. The first theoretical implication lies in theorizing the trickle-down nature of leaders’ VWGB. Specifically, we are among the first to theorize and propose a model in which leaders’ VWGB trickles down to employees in the organization (Kim et al., 2017, 2019). In doing so, we contribute to the existing literature by conceptualizing environmental behaviors at the leader level as a form of modeling a specific work behavior (i.e., VWGB) for followers that can boost pro-environmental behavior at the employee level. Although previous studies showed a positive impact of leaders’ green-related behaviors on desirable outcomes among employees in the workplace (Robertson and Barling, 2013; Chen et al., 2014; Mittal and Dhar, 2016), they failed to directly and rigorously test specifically for a trickle-down effect of leaders’ VWGB. Thus, we extend the green leadership research stream on the cascading effect to include leaders’ VWGB as perceived by employees, revealing that leaders’ VWGB flows down the hierarchy from higher-level leaders to lower-level employees in organizations.

Another contribution is our exploration of the mechanism through which the effects of leaders’ VWGB trickle down to employees’ pro-environmental behaviors. In doing so, we respond to scholars’ calls for more investigations to open the black box of the influences of green leadership (Fernández et al., 2006; Renwick et al., 2013; Norton et al., 2015) by providing empirical evidence of employees’ environmental self-identity as a mediator. Moreover, by applying social identity theory, our results highlight the salience of social identity as a prominent channel through which leaders’ green-related behaviors such as VWGB as perceived by employees can affect followers’ green outputs; in doing so, we extend the application of social identity theory in the participative leadership literature (Ellemers et al., 2002; Christensen et al., 2004; Chen, 2011; Chang and Chen, 2013). Meanwhile, our findings respond to scholars’ arguments that leaders are essential for conveying a green mindset to other organizational members who develop a green self-identity in the workplace.

Finally, we extend the current understanding of how employees’ green climate perceptions play an essential role in boundary conditioning the trickle-down effect of leaders’ VWGB. That is, the effect of leaders’ VWGB as perceived by employees can be strongly triggered when employees perceive a green climate in their workplace. These findings on the contingent role of green climate perceptions demonstrate the critical role of the organizational climate and norms related to green issues in influencing employees’ environmental behaviors (Zientara and Zamojska, 2018). Consistent with situational strength theory (Kerr and Jermier, 1978), our findings empirically support the arguments from the previous leadership literature that green climate, as a situational strength, can help employees embrace green behaviors and attitudes more fully (Dumont et al., 2017). In this vein, we make a unique contribution by investigating situational factors that are part of the influencing process connecting leaders’ VWGB as perceived by employees and employees’ green self-identity and pro-environmental behaviors as perceived by their leader.

### Practical Implications

Based on our findings in this empirical study, we suggest several practical implications. First, considering the beneficial influences of leaders’ VWGBs, organizations should pay attention to how they are encouraging VWGBs among managers and leaders. For example, organizations can select and promote leaders who engage in VWGBs during work hours and provide environmental training courses to managers. In addition, the human resources department can establish and implement environmental standards throughout the organization to help leaders highlight the pro-environmental characteristics of policies within firms. Meanwhile, leaders and managers are highly encouraged to realize their role as a green role model; that is, they should behave in an environmental manner and set an example of VWGB in the organization towards natural environment protection.

Furthermore, given the results that green climate perceptions act as a strengthening moderator, it is reasonable to conclude that organizations can benefit from creating a climate with a green orientation in which both leaders and followers are encouraged to engage in environmentally friendly behaviors and are rewarded for doing so. In this vein, we suggest that organizations encourage staff to not only participate in addressing environmental issues in the workplace but also develop environmental norms to foster a collective green environment.

### Limitations

The first limitation in this work concerns the generalizability of the sample. Since we collected data from hotels only, our findings should be most generalizable to other similar industries or organizations. For example, considering that environmental protection is becoming an international issue at an increasing rate among various industries, especially in the manufacturing section (Dangelico et al., 2017), leaders’ VWGB may facilitate green innovative outcomes among followers (Fernández et al., 2006); therefore, future researchers are encouraged to investigate whether leaders’ VWGB as perceived by employees exerts a trickle-down effect on followers’ pro-environmental behavior as perceived by their leader specifically in such workplaces.

Related to the first point, it is acknowledged that a selection of more heterogeneous samples in relation to the type of organizations and professional sectors would be appropriate; thus, it is highly recommended that future studies replicate our research findings by selecting more heterogeneous samples from various organizations in distinguished industries. In addition, although we controlled for variables such as employees’ genders and ages to preclude their potential influences in the proposed relationship, we failed to take other variables into consideration. Specifically, some scholars have suggested that the homogeneity of the selected institutions and some complementary context variables (e.g., environmental quality policies and hotel chains) may exert potential influences on employee outcomes in the hospitality literature (e.g., Chou, 2014). Thus, we encourage future research to follow this line of reasoning by exploring the potential influences. For example, because green human resource management practices can influence employees’ green involvement and behavior in the work environment (Pham et al., 2019), they might enable employees to perceive the support for and benefits from green practices and become more likely to participate voluntarily in green activities. As a result, employees would be more likely to follow these valued green principals towards producing green and environmental outputs.

Second, although we identified a trickle-down effect of leaders’ VWGB as perceived by employees on pro-environmental outcomes among employees, we did not examine the effect at the team level. Since employees generally work on teams, we suggest that future studies explore the implications of the trickle-down effect on team-level pro-environmental outcomes. For example, when a leader displays a VWGB, he/she tends to highlight collective interests that would encourage team members to work together on environmental issues for the green benefits of the whole team (Cai et al., 2020). Thus, the team’s pro-environmental work behaviors may increase.

The third limitation concerns the potential influences of group members on employees’ behavioral outcomes. Since previous studies have indicated that the interactions among team members within a group can exert an impact on a group’ working environment (e.g., Cai et al., 2020), we encourage future studies to take group members’ green-related attitudes and behaviors into consideration to further establish our research findings.

Finally, past literature has indicated the impacts of organizational structure and environmental strategy on employees’ pro-environmental outputs; however, these potential factors were not included in our study. Therefore, future investigations should focus on identifying these factors to further support our findings.

## Conclusion

Although previous studies have suggested the positive influences of leaders’ green behaviors such as VWGB as perceived by employees on followers’ desirable outcomes, little attention has been given to understanding whether, how, and when VWGB can exert a trickle-down influence from the leader level to the employee level. Accordingly, to fill these research gaps, the current study, drawing on social identity theory, explores leaders’ VWGB and shows how it can exert a trickle-down effect on employees’ pro-environmental behaviors as perceived by their leader through green self-identity and under the boundary condition of a green climate. The results show the positive trickle-down effect of leaders’ VWGB on employees’ pro-environmental behaviors as perceived by their leader (i.e., task-related pro-environmental behavior and proactive pro-environmental behavior) through increasing employees’ green self-identity. Moreover, we find that this mediation relationship is conditioned on the positive moderator of employees’ green climate perception.

## Data Availability Statement

The raw data supporting the conclusions of this article will be made available by the authors, without undue reservation.

## Ethics Statement

The studies involving human participants were reviewed and approved by the Vrije Universiteit Amsterdam Ethics Committee. The patients/participants provided their written informed consent to participate in this study.

## Author Contributions

JW and DC: conceptualization. CP and WC: methodology. WZ: software and data curation. CP and JL: validation. JW: formal analysis and project administration. SZ: investigation. DC: resources and supervision. JL and SZ: writing—original draft preparation. WC: visualization. All authors contributed to the article and approved the submitted version.

### Conflict of Interest

JW, WZ, CP, JL, and SZ were employed by company Anhui Ance Think Tank Consultancy Co., Ltd.

The remaining authors declare that the research was conducted in the absence of any commercial or financial relationships that could be construed as a potential conflict of interest.
